# Cerebellar Ataxia in the Setting of Hashimoto’s Thyroiditis: A Case Report

**DOI:** 10.7759/cureus.29840

**Published:** 2022-10-02

**Authors:** Sahil Sardana, Betsy Johnson, Wing Chung Cyrus Cheng, Diana M Montenegro, Peter G Bernad

**Affiliations:** 1 Neurology, Neurology Services, Inc., Washington, DC, USA; 2 Internal Medicine, Dayanand Medical College and Hospital, Ludhiana, IND; 3 Public Health, Northern Illinois University, DeKalb, USA; 4 Medicine and Therapeutics, The Chinese University of Hong Kong, Prince of Wales Hospital, Shatin, HKG; 5 General Surgery, California Institute of Behavioral Neurosciences & Psychology, Fairfield, USA; 6 Neurology, George Washington University Hospital, Washington, DC, USA

**Keywords:** anti thyroid antibodies, steroid-responsive encephalopathy associated with autoimmune thyroiditis, anti-tpo antibodies, hashimoto’s thyroiditis, hashimoto’s encephalopathy, cerebellar-ataxia

## Abstract

Hashimoto’s encephalopathy (HE) is a rare diagnosis with a heterogenous presentation. It may not be directly related to thyroid dysfunction as most patients are euthyroid when the symptoms start. There has been a lack of consensus building on the pathophysiology of HE, but most of the evidence points towards autoimmune vasculitis as the underlying process. HE can present as seizures, cognitive dysfunction, tremors, or stroke-like symptoms with focal neurological deficits. Cerebellar ataxia (motor incoordination due to dysfunction of the cerebellum) is seen in HE but is a rare occurrence.

The objective of the article was to present a case of cerebellar ataxia in a patient with Hashimoto’s thyroiditis. A 30-year-old previously healthy female presented with quickly progressive cerebellar ataxia, bilateral (B/L) limb weakness, and excessive tearing. She was found to have high titers of anti-TPO (anti-thyroid peroxidase) antibodies; a biopsy confirmed Hashimoto’s thyroiditis and a battery of negative tests excluding other causes of encephalopathy. Hence, confirming a diagnosis of HE. The patient was given glucocorticoids which relieved her symptoms. After being symptom-free for a few months, she relapsed and was unsuccessfully treated by the steroids. Upon this, she was given IV immunoglobulins, which helped achieve complete resolution. HE can be treated with immunotherapy, and most patients have a good prognosis, but some can have persistent neurological defects if left untreated or treatment is delayed. Relapses are common and may require a more extended treatment regimen.

## Introduction

The first neurological illness associated with Hashimoto's disease was reported by Brain et al. in 1966 [1]. Hashimoto's encephalopathy (HE) is associated with Hashimoto's thyroiditis and is a relapsing-remitting neurological disease with a varied presentation ranging from seizures, cognitive impairment, and stroke-like symptoms [2-3]. Cerebellar ataxia is rare as a presentation of HE [4]. Women are more affected than males, with the average age being 40 years [3]. HE is a diagnosis of exclusion, and other toxic, metabolic and infectious causes must be ruled out first [2, 5]. HE is treated with steroids, while encephalopathy associated with hypothyroidism is treated with thyroxine [6]. In this article, we present a case report of a patient who had a progressive cerebellar disease along with pseudo-bulbar symptoms associated with Hashimoto's thyroiditis in the setting of high anti-thyroid peroxidase (anti-TPO) antibodies. The patient was treated with several rounds of corticosteroids before she was put on IV immunoglobulins due to relapse.

## Case presentation

A 30-year-old previously healthy right-handed female had an initial presentation of sudden onset of tremors and loss of dexterity in bilateral (B/L) hands in 2019. Over the day, she developed slurred speech and weakness over B/L upper and lower limbs, with the symptoms worsening on the left side. Initial workup of blood tests, CT, and MRI done on the same day at the emergency department revealed no abnormalities. Her symptoms worsened, and she could not walk independently, and her speech became unintelligible when she was discharged three days later. During the neurology consultation two weeks later, she described ataxic symptoms of dysmetria, bradykinesia, movement incoordination, mild tingling sensations in her hands, and clumsy balance while sitting and standing. There were no problems with chewing, swallowing, or breathing and no abnormal body movements. She noticed blurred vision, tunnel vision, and nystagmus in B/L eyes several times a day, which resolved later. She started to tear excessively following the onset of the other symptoms, which is suggestive of pseudobulbar affect. She denied any diplopia, vertigo, hearing problems, psychiatric symptoms, memory changes, lack of concentration, or cognitive difficulties. She led an active lifestyle and was generally in good health except for subclinical hypothyroidism, diagnosed in 2014 when she had trouble conceiving her second child. She has been taking synthroid regularly since then. There is a family history of goiter and Grave's disease. There is no history of other chronic diseases or malignancy. The patient does not smoke or drink.

On physical examination, muscle tone was normal. Diminished power was noted on all four limbs. The finger-nose and heel-to-shin tests to assess the cerebellar function were dysmetric on the left side. The patient had intentional tremors, dysmetria, and dysdiadokokinesia and was unsteady upon standing up. Her tandem gait was abnormal. Romberg's test was negative. The reflexes on both upper limbs and lower limbs were brisk. The plantar reflex was down-going. There was no loss of pinprick or proprioceptive sensation. On examination of the eyes, there is no evidence of ophthalmoplegia, visual field defects, or nystagmus.

The patient's workup included various blood tests, spinal tap, EEG, MMSE, CT, and MRI of the brain to screen for infections, autoimmune diseases, structural abnormalities, or any underlying pathology. She tested negative for systemic lupus erythematosus (SLE) and multiple sclerosis. In view of the patient's past medical history of hypothyroidism, thyroid function test, thyroid antibody blood test, thyroid ultrasound, and thyroid biopsy were ordered. It was found that the patient had high titers of anti-TPO antibodies. A thyroid ultrasound revealed multiple nodules in the thyroid glands. The biopsy result showed that the patient had Hashimoto's thyroiditis. The clinical picture was suggestive of Hashimoto's thyroiditis with cerebellar dysfunction. The results of the investigations done are given in Table 1. The patient was started on a two-week course of methylprednisone. The symptoms improved as soon as day three after treatment was started but relapsed after the treatment was tapered off. As a result, the patient received three more courses of methylprednisone. She was also given dextromethorphan/quinidine for pseudobulbar symptoms. Following the last course of steroids, she enjoyed a symptom-free period for 18 months until recently, when she had hand tremors and slurred speech again. The symptoms were milder this time as the patient sought care early on and started on methyl prednisone within two days of symptom onset. However, complete remission of symptoms was not achieved despite the trial of three courses of steroids. She was prescribed intravenous immunoglobins (IVIG) but did not receive them initially due to insurance issues. In April 2022, she was started on IVIG, which resulted in the resolution of almost all the symptoms, including tearing and crying. She is also advised on physical therapy and occupational therapy. Currently, she is in remission with the next cycle of IVIG in one month.

**Table 1:** Summary of all relevant investigations done in this case. wo, without; w w/o, with and without; MMSE, Mini-Mental State Examination; EEG, electroencephalogram

Tests	Date	Result
CT head wo IV contrast	July 2019	No acute intracranial hemorrhage, midline shift, or mass effect, and no CT evidence of acute intracranial abnormality
MRI head wo IV contrast	August 2019	No acute intracranial abnormality. No infarct.
Ataxia, common repeat expansion evaluation	September 2019	Ruled out genetic variants associated with hereditary ataxia
Thyroid peroxidase antibody	January 2020	Elevated (158 IU/mL)
MRI brain w w/o contrast	February 2020	Unremarkable
Blood work	March 2020	Thyroid peroxidase antibody was elevated (127 IU/mL), Vitamin D was less (24.6 ng/mL).
MMSE	July 2021	Normal
EEG	July 2021	No epileptiform discharges
Thyroid peroxidase antibody	October 2021	Elevated (249 IU/mL)
Thyroid peroxidase antibody	February 2022	Elevated (254 IU/mL)
MRI brain	March 2022	Stable punctate region of susceptibility in the posterior right latera; aspect of the pons compared with brain MRI study dated February 2020

## Discussion

Hashimoto's encephalopathy is also known as steroid-responsive encephalopathy associated with autoimmune thyroiditis (SREAT). It has a heterogenous presentation ranging anywhere from stroke-like features, focal neurological deficits, seizures, or changes in the level of consciousness. At the time of presentation, most patients have normal thyroid function. Females are 10 times more affected than males, and it's relapsing in nature which could be a diagnostic sign [4].

Current literature has not been able to establish a well-defined pathological process for HE. One theory is the direct toxic effect of thyrotropin release hormone (TRH) on the brain. Another theory hypothesized the direct toxic effects of antithyroglobulin, anti-thyroid peroxidase autoantibodies, and circulating immune complexes (CICs) on the brain. But lack of association between HE and Abs(antibodies)/circulating immune complex (CIC) cerebrospinal fluid (CSF) levels in a study done by Ferracci et al. could not prove this theory. Forchetti et al. proposed that deposition of autoantibodies or immune complexes can cause interference in cerebral microvasculature, leading to HE. However, the latest studies have suggested vasculitis as the underlying pathological process. The same has been corroborated by CT angiography done in patients with HE [7].

Diagnosis of HE can only be achieved after excluding other causes of encephalopathy and seizures like space‐occupying lesions, Creutzfeldt‐Jakob disease, stroke, vasculitis, metabolic disorders, poisoning, and mental/psychiatric disorders. Brain MRI and CT, electroencephalogram (EEG), lumbar puncture, CSF examination, and blood laboratory testing are usually required to achieve the diagnosis by excluding the above differential diagnosis. Another important test for diagnosis is the presence of anti-thyroid antibodies, but their role in the disease process is yet to be established as the levels of antibodies do not correspond to the severity of the disease [8].

Table 2 showcases the criterion given by Graus et al. for the diagnosis of HE [9].

**Table 2:** Diagnostic criterion for HE formulated by Graus et al. CSF, cerebrospinal fluid; HE, Hashimoto's encephalopathy

	Diagnosis can be made when all six of the following criteria have been met
1.	Encephalopathy with seizures, myoclonus, hallucinations, or stroke-like episodes
2.	Subclinical or mild overt thyroid disease (usually hypothyroidism)
3.	Brain MRI normal or with non-specific abnormalities
4.	Presence of serum thyroid (thyroid peroxidase, thyroglobulin) antibodies
5.	Absence of well-characterized neuronal antibodies in serum and CSF
6.	Reasonable exclusion of alternative causes

Another vital evidence to suggest HE is the improvement with steroids and, therefore, the name SREAT. Table 3 lists the first-line and the second-line treatment options as suggested by Guo et al. in their study [8].

**Table 3:** Treatment of HE as suggested by Guo et al. [8] HE, Hashimoto's encephalopathy; IVIG, intravenous immunoglobulins

Treatment of HE	
First line	Second line
High dose glucocorticoids	Rituximab
IVIG	Cyclophosphamide
Plasma exchange	

## Conclusions

Our patient initially presented with predominantly cerebellar signs like tremors but quickly progressed to fully developed cerebellar ataxia, pseudobulbar signs, and B/L upper and lower limb weakness in the setting of Hashimoto's thyroiditis which was confirmed by high anti-TPO antibodies and a positive thyroid biopsy. She responded very well to methyl prednisone but relapsed as soon as steroids were tapered off. She received three more courses of methyl prednisone plus dextromethorphan/quinidine for pseudobulbar symptoms. After 18 months of being symptom-free, she relapsed again, but this time methyl prednisone could not achieve complete recovery; therefore, immunoglobulins were given, which resulted in the resolution of symptoms more effectively and completely. She is currently in remission.

Hashimoto's encephalopathy is an under-recognized disease and affects females multiple times more than males. To find out the exact pathologic process of HE more research is required. The diagnosis of HE is that of exclusion. The presence of anti-thyroid antibodies and exclusion of other differential diagnoses supports diagnosis in favor of HE, but there is insufficient evidence that levels of anti-thyroid antibodies have any correlation with the severity of the disease. In our view, anti-thyroid antibodies can be tested as a second or third line of investigation in suspected cases. Corticosteroids are the treatment of choice, but severely sick patients can also be treated with plasma exchange. An important takeaway is that even though the cases of HE are challenging to diagnose, the treatment is not that complex, so physicians should have a low threshold to check for HE in patients presenting with unexplained seizures, stroke-like symptoms, cerebellar ataxia, or cognitive impairment.
